# Multiplex Autoantibody Detection in Patients with Autoimmune Polyglandular Syndromes

**DOI:** 10.3390/ijms22115502

**Published:** 2021-05-23

**Authors:** Elena N. Savvateeva, Marina Yu. Yukina, Nurana F. Nuralieva, Marina A. Filippova, Dmitry A. Gryadunov, Ekaterina A. Troshina

**Affiliations:** 1 Center for Precision Genome Editing and Genetic Technologies for Biomedicine, Engelhardt Institute of Molecular Biology, Russian Academy of Sciences, 119991 Moscow, Russia; mafilippova@mail.ru (M.A.F.); grad@biochip.ru (D.A.G.); 2 Endocrinology Research Centre, Ministry of Health of Russia, 117036 Moscow, Russia; yukina.erc@yandex.ru (M.Y.Y.); nnurana@yandex.ru (N.F.N.); troshina@inbox.ru (E.A.T.)

**Keywords:** autoimmune polyglandular syndrome, autoantibodies, microarray, multiplex assay

## Abstract

The diagnosis of autoimmune polyglandular syndrome (APS) types 1/2 is difficult due to their rarity and nonspecific clinical manifestations. APS-1 development can be identified with assays for autoantibodies against cytokines, and APS-2 development with organ-specific antibodies. In this study, a microarray-based multiplex assay was proposed for simultaneous detection of both organ-specific (anti-21-OH, anti-GAD-65, anti-IA2, anti-ICA, anti-TG, and anti-TPO) and APS-1-specific (anti-IFN-ω, anti-IFN-α-2a, and anti-IL-22) autoantibodies. Herein, 206 serum samples from adult patients with APS-1, APS-2, isolated autoimmune endocrine pathologies or non-autoimmune endocrine pathologies and from healthy donors were analyzed. The prevalence of autoantibodies differed among the groups of healthy donors and patients with non-, mono- and multi-endocrine diseases. APS-1 patients were characterized by the presence of at least two specific autoantibodies (specificity 99.5%, sensitivity 100%). Furthermore, in 16 of the 18 patients, the APS-1 assay revealed triple positivity for autoantibodies against IFN-ω, IFN-α-2a and IL-22 (specificity 100%, sensitivity 88.9%). No anti-cytokine autoantibodies were found in the group of patients with non-APS-1 polyendocrine autoimmunity. The accuracy of the microarray-based assay compared to ELISA for organ-specific autoantibodies was 88.8–97.6%. This multiplex assay can be part of the strategy for diagnosing and predicting the development of APS.

## 1. Introduction

Autoimmune endocrinopathies can be grouped within autoimmune polyglandular syndromes (APSs). APSs are classified as rare monogenic APS-1 and more common polygenic types. Since APS types 2, 3 and 4 are characterized by a similar mechanism of development of the disease, a polygenic type of inheritance [1], the manifestation of endocrinopathies in most cases in adulthood, and the variability of combinations of syndromic components over time, most experts identify a single type of APS (APS type 2) [2].

APS-1, also known as autoimmune polyendocrinopathy-candidiasis-ectodermal dystrophy (APECED), is a rare monogenic disorder caused by mutations in the autoimmune regulatory (*AIRE*) gene. The classic triad components of APS-1 are chronic mucocutaneous candidiasis (CMC), hypoparathyroidism, and Addison’s disease (AD) [2]. The primary manifestations of APS-1 occur, as a rule, from the first year of life to 18 years. The criteria for the diagnosis of APS-1 are the presence of two of the three characteristic diseases, or one in the case of a disease in siblings. However, the classic diagnostic dyad covers only a small fraction of new cases. By the age of 5 years, the diagnostic dyad can be detected in 21% of patients, and the diagnostic triad can be detected in 3% of patients. The complete picture of the disease appears much later; 99% of 40-year-old patients have a diagnostic dyad, and 71% have a diagnostic triad [3]. The diagnosis of APS-1 can be confirmed by genetic testing, and more than 100 mutations in the *AIRE* gene have been described thus far [4].

Additional diagnostic criteria for APS-1 can be the identification of specific biomarkers—autoantibodies to interleukin 22 (IL-22) [5,6] and type I interferons [7]. Hence, Kisand et al. found neutralizing antibodies to IL-22 in APS-1 patients with CMC (in 91% of cases), while these antibodies were not identified in patients with CMC without a mutation in the *AIRE* gene, isolated hypoparathyroidism, isolated AD, APS-2, psoriasis, rheumatoid arthritis and other autoimmune diseases [6]. Since Meager et al. [7], using the method of neutralizing antiviral interferons in 2006, found a high titer of neutralizing immunoglobulin G to type I interferons in patients with APS-1, a number of highly specific tests have been developed and validated to detect such autoantibodies in patients with APS-1. Among them, analyses are based on ELISA [8], immunoprecipitation [9], radioligand binding assays [10], neutralization reactions of viruses in cell culture [11,12,13], HEK-blue cell assays [14], microarrays [15], and proteome-wide programmable phage display approaches [16].

The detection of autoantibodies to cytokines can be used to test relatives of patients with APS-1, as well as in the atypical course of APS, which is often found in clinical practice [2,17,18,19]. Thus, in some cases, in childhood, only “small” components of APS-1 appear, while the first main component of the disease manifests itself in adulthood [20].

Given that autoimmune endocrinopathies are often life-threatening conditions, their timely diagnosis is extremely important for both patients and their relatives. Organ-specific autoantibodies associated with organ-specific autoimmune diseases can be detected prior to the development of clinically overt disease [21,22]. The most common combinations of APS are autoimmune thyroid diseases (AITDs), type 1 diabetes (T1D) and AD [23]. The development of relevant diagnostic and screening protocols to identify these patients in a timely manner is warranted. Thus, it seems reasonable to perform antibody screening for thyroperoxidase (TPO) and thyroglobulin (TG), glutamic acid decarboxylase (GAD-65), islet cell cytoplasmic antigen (ICA), tyrosine phosphatase-like protein (IA2) and steroid 21 hydroxylase (21-OH) to reveal AITDs [24], T1D [25] and AD [26]. For example, in any patient with one of these organ-specific autoimmune diseases, determination of such antibodies should be performed, and if negative, repeated every few years [27,28].

Thus, for the purpose of differential diagnosis of APS, as well as the diagnosis of autoimmune endocrinopathies at both the manifest and latent stages, it is important to create a multiscreening system based on a one-step study of immunological markers—antibodies to cytokines and to target organ tissues. The aim of this work was to develop and evaluate a multiplex method for detecting APS-specific autoantibodies, including autoantibodies (auto-Abs) to interleukin 22, omega and alpha-2-a interferons (IFN-ω and IFN-α-2a) and organ-specific autoantibodies to 21-OH, GAD-65, IA2, ICA, TG, and TPO.

## 2. Results

### 2.1. Detection of Autoantibodies by the Microarray-Based Assay

**Figure 1:**
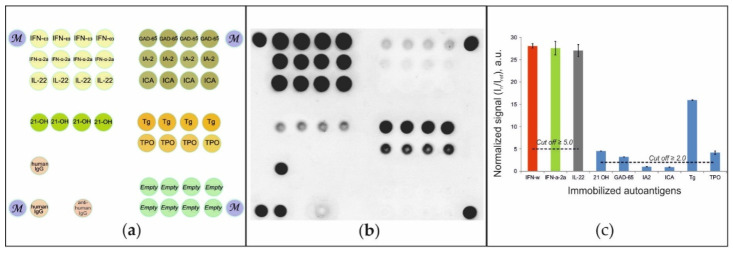
(**a**) Microarray configuration; (**b**) fluorescence image of the microarray; and (**c**) normalized signals from the microarray elements after assay of the serum sample from patient #49 with APS-1. Designations: IFN-ω—ntierferon omega; IFN-α-2a—interferon-alpha-2a; IL-22—interleukin 22; 21-OH—steroid 21 hydroxylase; GAD-65—glutamic acid decarboxylase 65 kDa; IA-2—tyrosine phosphatase-like autoantigen; ICA—islet cell autoantigen 1; TPO—thyroid peroxidase; TG—thyroglobulin; M—marker; human IgG—human immunoglobulin G; anti-human IgG—mouse anti-human immunoglobulin G; Empty—reference gel elements without immobilized protein.

To detect autoantibodies in patient serum, a hydrogel-based microarray with immobilized autoantigens was developed (Figure 1A). Diluted serum samples were applied to the microarray, and autoantibodies bound to immobilized autoantigens were detected using anti-species fluorescently labeled antibodies. Figure 1B shows a sample fluorescence image of a microarray after analysis of a serum sample from a patient diagnosed with APS-1. For this sample, positive signals indicating the presence of autoantibodies in blood serum were detected for groups of elements containing IFN-ω, IFN-α-2a and IL-22, as well as for groups of elements containing 21-OH, GAD-65, TG and TPO. Our statistics indicated that the positive signal should be at least 2.0-fold higher than the background signal I_ref_ with a standard deviation of 0.2. Thus, a 2.0-fold difference in intensity was used as the cut-off value for selecting positive signals. We also found that an increase in the threshold level for autoantibodies against IFN-ω, IFN-α-2a and IL-22 to I_n_/I_ref_ ≥ 5.0 allowed the exclusion of false positive results without loss of sensitivity. Figure 1C illustrates the values of normalized signals from groups of microarray elements: I_n_/_Iref_ ≥ 2.0 for autoantibodies against 21-OH, GAD-65, TG and TPO and exceeded the cut-off value (I_n_/I_ref_ ≥ 5.0) for autoantibodies against IFN-ω, IFN-α-2a and IL-22. The vertical bars show the scatter of the normalized signals within one group of elements.

### 2.2. Detection of Autoantibodies Against IFN-ω, IFN-α-2a and IL-22

**Figure 2:**
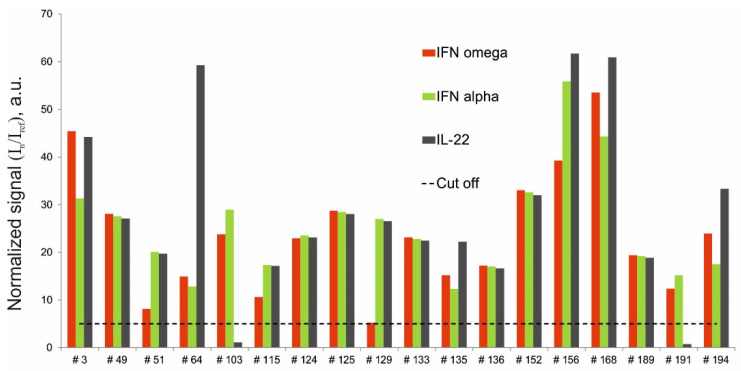
Normalized signals for autoantibodies against IFN-ω, IFN-α-2a and IL-22 in serum samples from APS-1 patients, as determined by the microarray-based assay.

Of the 206 samples analyzed, 18 were obtained from APS-1 patients (Table 1). The multiplex assay revealed triple positivity for autoantibodies against IFN-ω, IFN-α-2a and IL-22 in 16 of the 18 patients with APS-1. Two patients with APS-1 (#103 and #191) were only positive for autoantibodies against IFN-ω and IFN-α-2a. Additionally, notably, one patient (#129) was positive for autoantibodies against IFN-ω with a normalized signal (5.2) just above the cut-off value (Figure 2).

**Figure 3:**
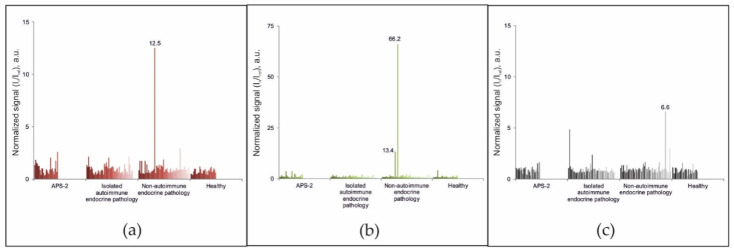
Normalized signals for autoantibodies against (**a**) IFN-ω; (**b**) IFN-α-2a; and (**c**) IL-22 (**c**) in serum samples from non-APS-1 patients, as determined by the microarray-based assay.

Among the 188 samples from patients in the comparison groups without diagnosed APS-1, exceeding the cut-off value for autoantibodies against either IFN-ω, IFN-α-2a or IL-22 was only revealed in 3 samples (Figure 3). The sample from patient #87 (female, 56 years old) with diagnosed primary hyperparathyroidism and multinodular goiter contained autoantibodies against IL-22. Analysis of the sample from patient #147 (female, 45 years old) with genetically proven multiple endocrine neoplasia type 1 (primary hyperparathyroidism; multiple insulinomas, non-functioning pancreatic tumors, and duodenal gastrinomas; lung carcinoids and multifocal hormonally inactive formations of both adrenal glands; and hyperprolactinemia) revealed autoantibodies against IFN-α-2a. The test sample from patient #56 showed the presence of autoantibodies against interferons IFN-ω and IFN-α-2a and the absence of autoantibodies against IL-22. This patient (male, 70 years old) was diagnosed with primary hypocorticism of a non-autoimmune origin due to newly diagnosed adrenal lymphoma, which was confirmed by computed tomography scanning of the adrenal glands as well as a normal level of autoantibodies against 21-OH. No other pathologies were identified. Genetic testing for the presence of mutations in the *AIRE* gene was not performed for these patients. Notably, triple positivity for autoantibodies against IFN-ω, IFN-α-2a, and IL-22 was not detected in any sample from patients in the comparison groups. Thus, using the condition of positivity for at least two of three autoantibodies (IFN-ω, IFN-α-2a, and IL-22), the diagnostic specificity and sensitivity of the microarray-based assay for APS-1 were 99.5% and 100%, respectively.

### 2.3. Detection of Organ-Specific Autoantibodies by Microarray-Based Assay and ELISA

The concordance rate between the multiplex microarray-based assay and individual ELISA kits for organ-specific autoantibodies against 21-OH, GAD-65, IA2, ICA, TG, and TPO was determined for all 206 samples (Table S1). To calculate the accuracy, precision and recall, the positive and negative ELISA results were used as true positive and true negative results, respectively (Table 2). The accuracy of the determination of organ-specific autoantibodies in the microarray-based assay compared to ELISA varied from 89.3% to 97.6%; thus, the developed assay can be used for screening positive organ-specific autoantibodies in serum.

### 2.4. Frequencies of Positive Autoantibodies in Serum Samples

**Figure 4:**
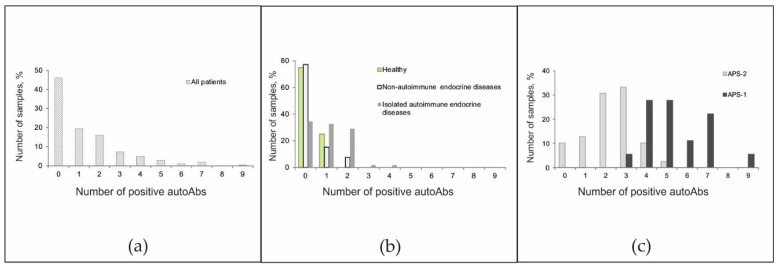
Frequencies of positive autoantibodies among (**a**) all patients; (**b**) healthy donors and patients without autoimmune polyendocrine syndromes; and (**c**) patients with autoimmune polyendocrine syndromes.

The frequencies of positive autoantibodies in serum samples from all patients were studied. Although in the overall cohort, the antibody detection rates were inversely proportional to the number of autoantibodies detected (Figure 4a), the pattern of the autoantibody detection rates was characteristic of individual groups. Among healthy patients, no more than one autoantibody was identified, and among patients with non-autoimmune endocrine diseases, no more than two autoantibodies were identified (Figure 4b). Among patients with isolated autoimmune disease, the proportions of patients with 0-, 1-, and 2-positive antibodies were nearly equal. However, a small number of patients had autoantibodies against 3–4 autoantigens. Most patients with APS-2 were characterized by the simultaneous presence of 2–3 autoantibodies (range, 0 to 5 autoantibodies). For APS-1, a bias towards a greater number of detected autoantibodies (range, 3 to 9 autoantibodies) was found (Figure 4c).

**Figure 5:**
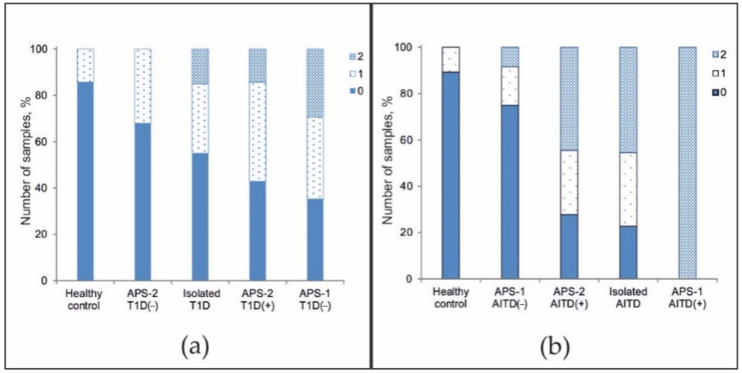
Frequency of positivity for organ-specific autoantibodies against (**a**) GAD-65, IA2, and ICA and against (**b**) TG and TPO among healthy controls and patients with APS-associated or isolated diseases. Designations: ‘+’—confirmed disease; ‘-’—absence of disease.

Additionally, we investigated the distribution of the frequency of detected organ-specific autoantibodies associated with T1D and AITDs in patients with isolated disease and in APS patients. At least one of three autoantibodies (GAD-65, IA2, or ICA) was detected in 14.3% of healthy patients (*n* = 28), in 33.3% of APS-2 patients without a T1D diagnosis (*n* = 25), in 30.0% of patients with T1D (*n* = 20), in 42.9% of APS-2 patients with a diagnosis of T1D (*n* = 14), and in 35.3% of APS-1 patients without a diagnosis of T1D (*n* = 17) (Figure 5a). In addition, two autoantibodies were detected simultaneously in patients with isolated T1D (15.0%), APS-2 patients with a diagnosis of T1D (14.3%), and patients with APS-1 without T1D (29.4%). Among all patients, only one patient with APS-1 and diagnosed with T1D (#115) was identified to be simultaneously positive for all three autoantibodies associated with T1D (data not shown).

At least one of two autoantibodies (TG or TPO) was detected in 10.7% of healthy patients (*n* = 28), 16.7% of APS-1 patients without AITDs (*n* = 12), 27.7% of APS-2 patients with a diagnosis of AITD (*n* = 36), and 31.8% of patients with isolated AITD (*n* = 22) (Figure 5b). In addition, two autoantibodies were detected in 8.3% of APS-1 patients who did not have AITDs, in almost half of APS-2 patients with a diagnosis of AITD (44.4%) or with isolated AITD (45.5%), and in all APS-1 patients with a diagnosis of AITD (*n* = 6). Only three APS-2 patients had no AITDs, though all three had autoantibodies against both TPO and TG (data not shown).

## 3. Discussion

In this study, a microarray-based multiplex assay was proposed for simultaneous detection of both organ-specific (anti-21-OH, anti-GAD-65, anti-IA2, anti-ICA, anti-Tg, and anti-TPO) and cytokine-specific (anti-IFN-ω, anti-IFN-α-2a, and anti-IL-22) autoantibodies that can be major biomarkers of autoimmune polyglandular syndromes. It is generally accepted that almost all APS-1 patients exhibit antibody reactivity to IFN-α and/or IFN-ω subtypes. However, these autoantibodies are also typical for patients with myasthenia gravis and thymoma [29]. Additionally, autoantibodies against type I interferons can be detected in patients with systemic lupus erythematosus (up to 10%), Sjogren’s syndrome (up to 8.7%), rheumatoid arthritis (up to 2%) [30], and incontinentia pigmenti [31].

The results of our study show that the proportion of people with autoantibodies against cytokines among patients without diagnosed APS-1 is small (1.6%, 3/188). Moreover, similar to Meloni et al. early [12], we found no anti-cytokine autoantibodies in a group of patients with non-APS-1 polyendocrine autoimmunity, whereas another study reported the opposite finding [32]. Although we did not assess the neutralizing ability of the identified autoantibodies against IFN-ω, IFN-α-2a and IL-22, the results obtained allowed us to correctly identify 18/18 patients with APS-1. Moreover, the detection of a characteristic signature of three autoantibodies (anti-IFN-ω + anti-IFN-α-2a + anti-IL-22) in a patient via this microarray allows the identification of patients with APS-1 with 100% specificity. However, the questions of whether this three-autoantibody signature is typical for patients with myasthenia gravis and thymoma and whether these autoantibodies can be detected in patients with other conditions remain open. Due to the rarity of APS-1, only a limited number of APS-1 patients were included in this study. A further study of a larger sampling of patients will make it possible to clarify the diagnostic characteristics of the developed method.

In two patients aged 18 (#103) and 44 (#191) with the classic clinical APS-1 triad and a genetically verified diagnosis, antibodies against IL-22 were not detected, as also described earlier [6]. No association of a negative result with remission of candidiasis and the duration of the history of fungal infection was found.

The formation of autoantibodies in patient #147 was presumably due to antiviral therapy, which he received for hepatitis C 17 years before inclusion in the study. This phenomenon is described in the literature [33,34]. Patient #56 denied therapy with IFN drugs. Data regarding the presence of myasthenia gravis and thymoma were not received. The development of anti-IFN antibodies in this patient may have been a consequence of lymphoma. Patient #87 was diagnosed with candidiasis of the urinary tract, which most likely explains the detection of antibodies against IL-22. Another feature of this patient was the location of the ectopic parathyroid gland tissue within the thymus. Thus, this patient could have developed antibodies as a result of thymic lesions, a characteristic of thymoma.

Since our study included patients over 18 years of age, the data obtained confirm the following results of previous studies: autoantibodies against type I interferons can be detected in adulthood, as well as in childhood [35], and titers of autoantibodies against IFN-ω, IFN-α-2a and IL-22 are almost always initially high and persist for decades after the onset of APS-1 [9]. Although the developed method is not intended to quantitatively measure the levels of autoantibodies, importantly, the levels of the recorded signals for autoantibodies against IFN-ω, IFN-α-2a and IL-22 were noticeably higher than those of signals from organ-specific autoantibodies, such as those against 21-OH, GAD-65, IA2, ICA, TG and TPO, consistent with previous data [15].

All patients included in the study were tested for the presence of organ-specific autoantibodies against 21-OH, GAD-65, IA2, ICA, TG and TPO. Since APS-2, like APS-1, rarely initially presents with two or more diseases simultaneously, and the complete clinical presentation of the syndrome can manifest gradually over many years, such testing is important for detecting a hidden autoimmune process, since autoantibodies can be detected before the manifestation of the disease.

When organ-specific autoantibodies were detected using microarrays, the following features were revealed: the number of positive autoantibodies detected increased from 0 to 9 across the row “healthy controls–patients with non-autoimmune endocrine diseases–patients with isolated autoimmune disease–APS-2 patients–APS-1 patients”. Thus, although organ-specific autoantibodies are not highly specific, detection of two or more autoantibodies in a patient most likely indicates an autoimmune disease.

The observed distribution of the frequency of detected autoantibodies associated with T1D was interesting. Notably, no patient with isolated T1D or T1D associated with APS-2 was simultaneously positive for one set of three antibodies (anti-GAD, anti-ICA, and anti-IA2). Only one patient in the entire cohort (*n* = 206) was positive for the three diabetes-associated autoantibodies; this patient (#115) was also the only one with T1D among the APS-1 patients. Although T1D develops in a minority of APS-1 patients, many APS-1 patients may have autoantibodies against GAD, even in the absence of diabetes [36]. Moreover, in patients with APS-1, these antibodies recognize different GAD epitopes than they recognize in patients with typical isolated T1D [37]. We found an absence of autoantibodies against GAD, ICA and IA2 in more than half of patients with longstanding T1D, which also confirms findings from previous studies [38].

Organ-specific autoantibodies can also be detected in healthy populations [39,40]. In our cohort of healthy donors (*n* = 28; 79% female, 21% male), a remarkably high percentage of organ-specific autoantibodies was found, as follows: 17.9% by ELISA and 25% by microarray. Positivity for autoantibodies against ICA was found in three patients, positivity for autoantibodies against GAD-65 was found in one patient by the microarray-based assay, and these findings were confirmed by the ELISA results. Additionally, the microarray-based assay identified three patients with positive autoantibodies against TPO, although these antibodies were not detected by ELISA. Among these patients, two underwent ultrasound examination of the thyroid gland, which revealed signs of AITD.

Thus, although the determination of autoantibodies plays the role of auxiliary rather than absolute diagnostic criteria, the detection of circulating autoantibodies, especially more than one, can be a highly effective tool to uncover hidden autoimmunity.

Recently, Bastard et al. identified high titers of neutralizing autoantibodies against type I IFN-α2 and IFN-ω in approximately 10% of patients with life-threatening COVID-19 pneumonia [31]. Moreover, these autoantibodies were detected in only 4 of 1227 (0.33%) healthy donors and in none of the 663 patients with asymptomatic or mild SARS-CoV-2 infection. Autoantibodies against IFN-ω and IFN-α-2 preceded infection with SARS-CoV-2 and were considered to be the cause of severe illness in these patients. Facing this finding, the method developed herein can be used for the additional assessment of the level of autoantibodies against type I IFNs in patients infected with SARS-CoV-2.

## 4. Materials and Methods

### 4.1. Clinical Data and Serum Samples

The study included the following blood serum samples from 206 patients aged 18–88 years: APS-1 (*n* = 18), APS-2 (*n* = 39), isolated autoimmune endocrine pathology (*n* = 50), non-autoimmune endocrine pathology (*n* = 71), and healthy donors (*n* = 28). Isolated autoimmune endocrinological pathologies included type 1 diabetes mellitus (*n* = 21), autoimmune thyroiditis (*n* = 12), Graves’ disease (*n* = 14), hypergonadotropic hypogonadism (autoimmune oophoritis) (*n* = 8), and Addison’s disease (*n* = 6). Non-autoimmune endocrine pathologies were presented by type 2 diabetes (*n* = 19), hyperparathyroidism (*n* = 12), and non-autoimmune thyroid diseases (*n* = 12).

In all cases, the diagnosis was based on medical history tracking, the following are results of hormonal blood tests and additional criteria:

- APS-1: classic clinical triad and/or mutation in the *AIRE* gene. The identification of mutations in the *AIRE* gene by Sanger sequencing was performed as described earlier [14,41];
- Hypergonadotropic hypogonadism of autoimmune genesis: the acquired form and combination with any autoimmune disease, antibody carrier and/or signs of autoimmune damage according to ultrasound of the thyroid gland were excluded;
- Primary autoimmune adrenal insufficiency: the acquired form is excluded, antibodies to 21-OH are increased;
- Autoimmune diabetes mellitus: the onset of the disease from a young age, within APS-2 and/or an increased level of antibodies to GAD, ZnT8, IA2, IAA and/or ICA;
- Autoimmune thyroiditis: Elevated levels of antibodies to TPO, TG and/or TSHR; signs of autoimmune lesion on thyroid ultrasound and/or medical history (in the case of Graves’ disease);
- Non-autoimmune hypergonadotropic hypogonadism: intact ovaries, testes; lack of concomitant autoimmune diseases;
- Non-autoimmune adrenal insufficiency: intact adrenal glands, normal level of antibodies to 21-OH; lack of concomitant autoimmune diseases;
- Non-autoimmune thyroid disease: normal level of antibodies to TPO, TG, and/or TSHR; no signs of autoimmune damage according to ultrasound of the thyroid gland; intact thyroid gland; lack of concomitant autoimmune diseases;
- Non-autoimmune diabetes mellitus: normal levels of antibodies to GAD, ZnT8, IA2, IAA, and/or ICA; intact pancreas; lack of concomitant autoimmune diseases;
- Non-autoimmune parathyroid disease: absence of concomitant autoimmune diseases and intact parathyroid gland;
- Healthy persons: absence of endocrine (autoimmune and non-autoimmune) pathology (according to the survey).

The characteristics of the study participants are shown in Table S1. Serum samples from patients were stored at −80 °C.

### 4.2. ELISAs for Detection of Autoantibodies

All serum samples were measured by ELISA to detect autoantibodies to 21-OH (BioVendor, Czech Republic), GAD (Euroimmun AG, Lübeck, Germany), IA2 (Medipan Gmbh, Berlin, Germany), ICA (Medipan Gmbh, Berlin, Germany), TPO (Abbott Laboratories, USA), and TG (Roche Diagnostics, Basel, Switzerland). For autoantibodies against IFN-α2 and IFN–ω and IL-22, the comparison method was not available.

### 4.3. Microarray Design and Manufacturing

Molecular profiling of autoantibodies in serum samples was performed using hydrogel-based low-density microarrays [42]. Preparation of surfaces, mixture of gel monomers, polymerization and blocking of microarrays were carried out as described earlier [43]. The diameter of the gel elements was 150 ± 20 µm, and the distance between the elements was 300 µm. Each antigen (Table S2 Microarray-immobilized autoantigens) was immobilized in four repetitions to improve the reproducibility of the assay results (Figure 1A). The microarray also included eight empty elements without immobilized proteins, marker elements and control elements for detecting antibodies. The list of immobilized antigens and their concentrations are given in Table S2.

### 4.4. Fluorescent Antibody Labeling

For fluorescent antibody labeling, 1 μL of N-hydroxysuccinimide ester Cy5 (GE Healthcare, Chicago, IL, USA) in N,N-dimethylformamide (Sigma-Aldrich, St. Louis, MO, USA) (10 mg/mL) was added to 50 μL of a solution of anti-human IgG (F(ab’) 2-goat anti-human IgG Fc gamma secondary antibody (Invitrogen, Carlsbad, CA, USA) in bicarbonate buffer (Sigma-Aldrich, St. Louis, MO, USA) (1 mg/mL). The reaction was carried out for 1 h (22 °C; 550 rpm). The target product was isolated by gel filtration on a spin column containing Sephadex G-25 Coarse (GE Healthcare, Chicago, IL, USA) in PBS (Sigma-Aldrich, St. Louis, MO, USA).

### 4.5. Microarray Hybridization and Washing

Blood serum samples from patients were diluted 1:100 with 100 mM Tris-HCl buffer (Merc, Kenilworth, NJ, USA) containing 0.1% Triton X-100 (Sigma-Aldrich, St. Louis, MO, USA) and applied to the microarray elements (120 μL). After incubation (usually overnight, 37 °C), intermediate washing (PBS with 0.01% Tween 20 (Sigma-Aldrich, St. Louis, MO, USA), 20 min), rinsing and drying, the microarrays were treated with fluorescently labeled anti-species antibodies (5 μg/mL; F(ab’) 2-goat anti-human IgG-Cy5; 50 μL) in PBS buffer with 0.14% polyvinyl alcohol (Sigma-Aldrich, St. Louis, MO, USA) (50 kDa) and 0.14% polyvinylpyrrolidone (Sigma-Aldrich, St. Louis, MO, USA) (360 kDa). After incubation (30 min, 37 °C), the microarrays were washed (PBS with 0.01% Tween 20, 30 min), rinsed with H_2_O, and dried by centrifugation.

### 4.6. Analysis of Fluorescence and Interpretation of Results

Fluorescence images of the microarrays were acquired using a laser-excited analyser developed at the Engelhardt Institute of Molecular Biology (EIMB, Russia, Moscow) [44]. Measurement of microarray fluorescence was carried out using the ImaGel Studio software (EIMB). For better reproducibility for each group (n) of four elements with the same antigens, the resulting I_n_ signal value was calculated as the median of the four corresponding fluorescence signal values [43]. The variation coefficient of the signals within one group of elements with the same antigen, i.e., for each data point, did not exceed 15%. In a group of eight elements without immobilized proteins, the resulting value of the fluorescence signal I_ref_ was calculated as the median of the 8 corresponding fluorescence signal values. The cut-off values for selecting positive signals were I_n_/I_ref_ ≥ 5.0 for autoantibodies against IFN-ω, IFN-α-2a, and IL-22 and I_n_/I_ref_ ≥ 2.0 for autoantibodies against 21-OH, GAD-65, IA2, ICA, TG and TPO.

## 5. Conclusions

Differential diagnosis of APS is especially relevant in patients with atypical variations in the disease course. Screening for polyglandular autoimmunity in patients with isolated autoimmune disease, patients with APS and first-degree relatives of APS patients is significant for timely diagnosis at an early stage and essential to prevent the development of life-threatening conditions during clinical manifestation. A microarray-based multiplex autoantibody assay was developed, combining simultaneous detection of both APS-1-specific autoantibodies against type I IFNs and IL-22 and organ-specific autoantibodies against 21-OH, GAD-65, IA2, ICA, TG, and TPO. This assay allows the detection of autoantibodies associated with APS-1, AD, T1D and AITDs. The microarray revealed a signature of three positive autoantibodies against IFN-ω, IFN-α and IL-22 in 89% of APS-1 patients. No anti-cytokine autoantibodies were found in patients with non-APS-1 autoimmune polyendocrine syndromes. The prevalence of autoantibodies differed among the groups of healthy controls and patients with non-autoimmune endocrine diseases, patients with isolated autoimmune endocrine diseases and APS patients. The developed assay can be a useful additional tool in the diagnosis and prognosis of diseases constituting autoimmune polyglandular syndromes.

## Figures and Tables

**Table 1:** APS-1 Patients.

#	Age	Sex	CMC	CHP	AD	Comorbidity	AIRE Mutations
3	49	m	+	+	+	-	R257X/R257X
49	20	f	+	+	+	HH, AIT, VIT, DA, malabsorption syndrome, enamel hypoplasia	R257X/not found
51	18	f	+	+	+	HH	R257X/R257X
64	29	f	+	+	+	HH, atrophic gastritis, cataract	p.R257*/p.W78R
103	18	f	+	+	+	HH	R257X/R257X
115	30	f	+	+	+	AIT, T1D, DA, atrophic gastritis	R257X/R257X
124	18	m	+	-	+	Megaloblastic anemia, malabsorption syndrome, enamel hypoplasia, splenic hypoplasia	R257X/A58 V
125	45	f	+	+	+	HH, AIT, VIT, atrophic gastroduodenitis, cataract, corneal dystrophy	No genetic study was performed
129	45	m	+	+	+	AA	No genetic study was performed
133	27	m	+	-	+	AIT, DA, autoimmune fibrosing alveolitis	No genetic study was performed
135	30	f	+	+	+	HH, VIT, malabsorption syndrome, corneal dystrophy, ptosis, asplenia, atrophic gastroduodenitis, autoimmune hepatitis	R257X/R257X
136	28	f	+	+	+	HH, corneal dystrophy, atrophic gastritis	R257X/c.931delT
152	27	f	+	+	+	HH, AA, DA, enamel hypoplasia, tubulointerstitial nephritis	R257X/not found
156	36	m	+	+	+	-	No genetic study was performed
168	32	f	+	+	+	HH, AIT, DA, VIT	R257X/R257X
189	31	f	+	+	+	HH, atrophic gastroduodenitis malabsorption syndrome, enamel hypoplasia, retinitis pigmentosa, cataract, heterotropia	R257X/R257X
191	44	f	+	+	+	HH	R257X/R257X
194	25	f	+	+	+	HH, AIT, atrophic gastritis	R257X/c.821delG

Abbreviations: CMC—chronic mucocutaneous candidiasis; CHP—chronic hypoparathyroidism; AD—Addison’s disease; HH—hypergonadotropic hypogonadism (autoimmune oophoritis); AIT—autoimmune thyroiditis; T1D—type 1 diabetes; AA—alopecia areata; DA—diffuse alopecia; VIT—vitiligo. Designations: ‘#’—patient number; ‘+’—confirmed disease; p. R257*—substitution nonsense.

**Table 2:** Comparison of the accuracy of the microarray-based assay with ELISA for the detection of organ-specific autoantibodies.

AuAbs to:	ELISA Positive/Microarray Positive	ELISA Negative/Microarray Negative	ELISA Negative/Microarray Positive	ELISA Positive/Microarray Negative	Accuracy, %	Precision	Recall
21-OH	154	30	9	13	89.3	0.94	0.92
GAD-65	184	16	2	4	97.1	0.99	0.98
IA2	184	17	0	5	97.6	1.00	0.97
ICA	178	18	8	2	95.1	0.96	0.99
TG	160	35	8	3	94.7	0.95	0.98
TPO	126	57	14	9	88.8	0.90	0.93
Total	986	173	41	36	93.8	0.96	0.96

## Data Availability

The data presented in this study are available in the Supplementary Material (Table S1).

## Supplementary Materials

The following are available online at https://www.mdpi.com/article/10.3390/ijms22115502/s1, Table S1: Patient characteristics and results of testing serum samples for the presence of autoantibodies against IFN-ω, IFN-α-2a, IL-22, 21-OH, GAD-65, IA2, ICA, TG, and TPO using microarray assay and ELISA kits, Table S2: List of microarray-immobilized autoantigens.
