# Supplementary material for: Multiplex Autoantibody Detection in Patients with Autoimmune Polyglandular Syndromes
Source: Int J Mol Sci. 2021 May 23;22(11):5502. doi: 10.3390/ijms22115502 (PMC8197071; doi:10.3390/ijms22115502)
Supplement: Supplementary file 1 [file ijms-22-05502-s001.zip › ijms-1218029-supplementary/Savvateeva et al Table S2.pdf]

Table S2 Biochip-immobilized autoantigens

Disease	Abbreviation	Protein	#	C protein, µg in 1 ml of gel	Company
APS-1	IFN-ω	Interferon omega	300-02J	300	PeproTech, USA
	IFN-α-2a	Interferon-alpha	11100-1	300	PBL Assay Science, USA
	IL-22	Interleukin 22	200-22	300	PeproTech, USA
Addison disease	21OH	Steroid 21 hydroxylase	ab225641	600	Abcam, UK
Type 1 diabetes mellitus	GAD65	Glutamic acid decarboxylase	228-20881-2	111	RayBiotech, USA
	IA-2	Tyrosine phosphatase like autoantigen	ab42590	60	Abcam, UK
	ICA1	Islet cell autoantigen 1	ls-g57192	100	LifeSpan BioSciences (USA)
Autoimmune thyroid diseases	TPO	Thyroid peroxidase	TPO rec	300	Bialexa, Russia,
	Tg	Thyroglobulin	TG naiv	300	Bialexa, Russia,
